# The Impact of Selected Ingredients on the Predicted Glycemic Index and Technological Properties of Bread

**DOI:** 10.3390/foods13162488

**Published:** 2024-08-08

**Authors:** Ilaria Pasqualoni, Roberta Tolve, Barbara Simonato, Federico Bianchi

**Affiliations:** Department of Biotechnology, University of Verona, Strada Le Grazie 15, 37134 Verona, Italy; ilaria.pasqualoni@univr.it (I.P.); roberta.tolve@univr.it (R.T.); federico.bianchi_02@univr.it (F.B.)

**Keywords:** low glycemic index, chickpea flour, dietary fiber, resistant starches, red chicory by-product powder

## Abstract

Bread, a staple food consumed worldwide, plays a pivotal role in nutrition. Nevertheless, it is to be underlined that white bread is classified as a high glycemic index food, and its frequent consumption can lead to rapid increases in blood glucose, potentially causing metabolic stress and contributing to insulin resistance and type 2 diabetes. So, there is a growing interest in bread formulations with ingredients that can lower its GI. With this view, bread was formulated, substituting wheat with chickpea flour, red chicory powder, and three distinct types of resistant starch. The results showed the different resistant starches’ impacts on the glycemic index reduction. Specifically, chemically modified tapioca RS IV produced a bread formulation with a low predicted glycemic index (pGI < 55). Retrograded starch from tapioca (RS III) allows the bread to reach a pGI value of 55, the upper value for classifying a food as low pGI. The retrograded starch from corn (RS III) allows a decrease in the bread’s glycemic index, but the product is still classified as ‘high pGI’ (>70). Moreover, the addition of by-products rich in polyphenols contributes to a lowering of the pGI. Concerning the technological parameters, the outcome revealed an increase in the moisture content across all the newly formulated samples compared to the control. At the same time, the volume and specific volume showed a decrease. The newly formulated samples exhibited a higher baking loss, particularly when incorporating resistant starch, which increased the hardness and chewiness with decreased cohesiveness. In conclusion, incorporating chickpea flour, red chicory powder, and tapioca-resistant starch (RS III and IV) offers a promising strategy for producing high-fiber bread with a low glycemic index, catering to health-conscious consumers.

## 1. Introduction

Bread plays a pivotal role in the longstanding dietary traditions of the Mediterranean region, serving as a cornerstone in culinary practices. Traditionally, bread is made with gluten-rich wheat flour, contributing to its distinctive texture and palatability. Gluten’s structure is maintained by non-covalent interactions (hydrogen bonds, ionic bonds, hydrophobic interactions) and a small proportion of disulfide-type covalent bonds (about 2%). These interactions are crucial for protein aggregation, influencing the unique properties of commercial products. Wheat gluten’s characteristics derive from gliadins (providing viscosity) and glutenins (providing elasticity). Gliadins, monomeric proteins with molecular weights between 3 and 8 × 10^4^ Da, contain intra-chain disulfide bridges, while glutenins, polymeric proteins with molecular weights between 10^5^ and 10^7^ Da, have both intra- and inter-chain disulfide bonds, contributing to gluten’s rigidity and elasticity [1]. Wheat bread is typically classified as a high glycemic index food. The glycemic index (GI) measures how quickly a food can raise blood glucose levels and reflects the quality of carbohydrates in the diet. Specifically, the rate of enzymatic digestion of starch into glucose may vary from rapid to slow. Starch resistant to enzymatic hydrolysis is also known [2]. Frequent consumption of high-GI foods can lead to rapid increases in blood glucose, potentially causing metabolic stress and contributing to insulin resistance and type 2 diabetes [3]. Nowadays, increasing consumer awareness about health and wellness is transforming the food industry. As a result, there is a growing interest in bread formulations with ingredients that can lower its GI, such as different types of legume flours, dietary fibers (DF), proteins, and phenolic compounds that can enhance the bread’s nutritional profile [4,5]. DF contributes to various physiological effects, including decreased intestinal transit time, increased stool bulk, reduced postprandial blood glucose and insulin levels, gastric acid buffering, and the prevention of constipation [6,7].

Incorporating DF into food products offers various health benefits and functional attributes [8]. An ample spectrum of fiber can be selected to enrich foods; among them, resistant starches (RSs) have been evaluated and considered in the present study. 

RSs appeared to be a sensible alternative for developing functional bread and other starch-based foods due to their ability to reduce the caloric value of food products and to decrease the predicted glycemic index. RSs, being non-digestible, are fermented in the colon by the microbiota to produce short-chain fatty acids like acetic, propionic, and butyric acids, which favorably select intestinal microflora; reduce cholesterol, triglycerides, and urea levels; and prevent gut cancer [9]. RSs are classified into five types: physically inaccessible starch in undamaged plant cells (RS I), raw starch from certain plants like potatoes (RS II), retrograded starch forming water-insoluble structures (RS III), chemically or physically modified starch (RS IV), and amylose-lipid complexes (RS V) [10]. 

In addition to DF, phenolic compounds (PCs) can modulate starch digestibility by directly inhibiting α-glycosidase digestive enzymes and/or forming complexes with starch [11]. In this view, enhancing the nutritional profile of functional bread using red chicory by-products rich in PCs and DF aligns with the EU’s Green Deal initiatives [12,13]. 

Finally, chickpea flour (CPF), similar to other legumes, is a rich source of high-quality proteins and dietary fiber, which can help lower the glycemic response. Several factors contribute to this, including the rigidity of cotyledonary cell walls, legume starches’ low enzyme susceptibility, and polyphenols and other α-amylase inhibitors [14]. Thus, replacing wheat flour with chickpea flour can reduce the GI of wheat bread [15]. It is essential to highlight that including various ingredients, at the expense of wheat flour and its gluten content, also impacts the bread’s technological characteristics. While many researchers have assessed the nutritional benefits of bread supplemented with resistant starch, agrifood by-products rich in phenolic compounds, and legume flour, none have explicitly focused on how these ingredients’ single and simultaneous presence impact the resulting bread’s technological and glycemic index characteristics. 

This study aimed to understand the effect of red chicory powder, chickpea flour, and resistant starches on bread glycemic index reduction and its technological properties.

## 2. Materials and Methods

### 2.1. Ingredients and Breadmaking

**Figure 1:**
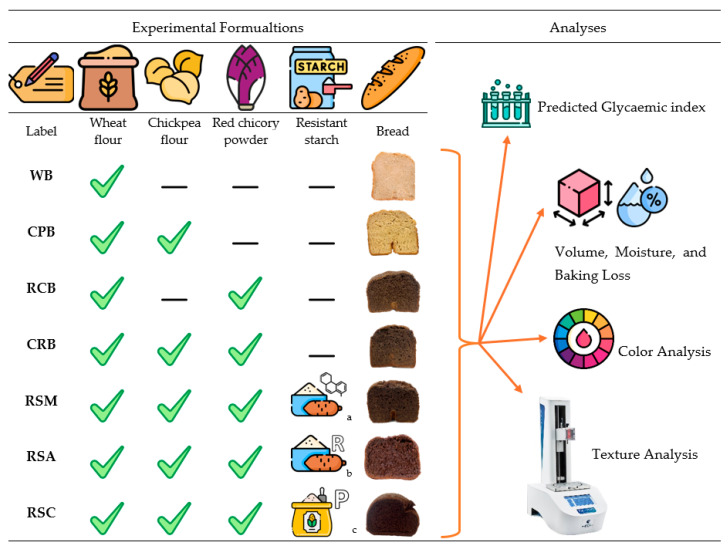
Summary of the experimental bread formulations and analyses performed in this study. a chemically modified tapioca resistant starch. b retrograded tapioca resistant starch. c pregelatinized corn resistant starch.

Common white wheat flour was kindly supplied by Macinazione Lendinara SpA (Arcole, Italy). The wheat flour label detailed the following composition: 343 kcal/100 g, fat 1.2 g/100 g, total carbohydrates 70 g/100 g, protein 12 g/100 g, and dietary fiber 2.3 g/100 g. Red Chicory leaves (*Cichorium intybus* L.) were provided by GEOFUR, a local consortium of Verona (Legnago, Italy). The leaves were dried in an oven at 50 °C for 16 h, ground with Grindomix GM 200 (Retsch, Haan, Germany), and went through a sieve with meshes of 0.2 mm to achieve a fine powder that has the following nutritional characteristics: 240 kcal/100 g, total carbohydrates 23.5 g/100 g, fat 3.3 g/100 g, fiber 27.4 g/100 g, and protein 15.8 g/100 g. The chickpea flour was purchased from Molino Rossetto (Padova, Italy), with the following composition: 355 kcal/100 g, fat 6.6 g/100 g, total carbohydrates 4.1 g/100 g, fiber 9 g/100 g, protein 21 g/100 g, and salt 0.03 g/100 g. Gluten, RSs, and guar gum were provided by ABS Food srl (Padova, Italy), and other ingredients such as sugar, salt, and dried yeast were purchased from a local market. The breads were prepared with a commercial breadmaking machine (Panexpress 750 Metal, model 0132/00—Ariete, Trieste, Italy). Doughs were mixed with a blender (Kenwood, Chef XL KVL4110S, Treviso, Italy), fermented for 3 h at room temperature, and then cooked at 170 °C for 2 h. The overview of the experimental bread formulations and analyses are reported in Figure 1, while the sample formulations expressed as percentages are reported in Table 1. 

### 2.2. Bread Hydrolysis Index and Predicted Glycemic Index

The hydrolysis index was determined following the methodology of Bianchi et al. [13]. One hundred milligrams of bread was incubated in glass vials at 37 °C. During the incubation, 4 mL of maleic buffer (pH 6) containing 40 mg of pancreatic amylase (3000 U/mg) and 4 mL of amyloglucosidase solution (300 U/mL) from Megazyme (Megazyme, Wicklow, Ireland) were added. The reaction was halted at 0, 30, 60, 120, and 180 min with 4 mL of pure ethanol introduction; the solution was then centrifuged at 1500× *g* for 10 min. The assessment of D-glucose was determined spectrophotometrically at 510 nm using a glucose oxidase kit (GOPOD, Megazyme, Wicklow, Ireland). The hydrolysis index was defined as the percentage ratio of the area under the curve representing pasta sample hydrolysis (0–180 min) to the area under the curve of a reference white bread. The pGI was calculated according to Granfeldt et al. [16] using a specific Formula (1).

(1) pGI=8.198+0.862×HI

### 2.3. Bread Quality Characteristics

#### 2.3.1. Moisture Content, Volume, Specific Volume, and Baking Loss

The moisture content was measured using the AACC method 44-15A [17]. The bread volume was determined by seed displacement [17], and the specific volume (cm^3^/g) was determined by dividing the volume by the weight of the samples. The baking loss (BL) was determined as the difference in mass between the dough and the baked loaves [7].

#### 2.3.2. Texture Profile Analysis of Bread

The texture profile analysis was performed using a TA-XT plus Texture Analyser (TX-700, Lamy Rheology, Champagne au Mont d’Or, France) with a 5 kg load cell to determine the hardness, cohesiveness, and chewiness. As described by Tolve et al. [7], two slices of bread (20 mm × 20 mm) were overlapped and subjected to a double compression using a metal cylindrical probe (25 mm diameter), operating at a speed of 1 mm/s with a deformation percentage of 50%. Hardness was calculated as the maximum force of the 1st cycle of compression, cohesiveness was the ratio of the work of the first compression and the second, and chewiness was a product of hardness, cohesiveness, and springiness, where springiness was the ratio between the distance of the 2nd and 1st compression.

#### 2.3.3. Color Analysis

The color was measured by a reflectance colorimeter (illuminant D65) (Minolta Chroma meter CR-300, Osaka, Japan) based on the color system CIE—L* a* b*. Analyses were performed at five different points within the crumb and the crust area. 

### 2.4. Statistical Analysis

The analyses were carried out in triplicate. All data are expressed as the mean values ± standard deviation. XLSTAT (Version 2406 Build 16.0.17726.20078, Addinsoft, Paris, France) was used for analyzing the data using the analysis of variance (ANOVA) with the post hoc Tukey’s test at *p* < 0.05.

## 3. Results and Discussion

### 3.1. Predicted Glycemic Index of Breads

**Figure 2:**
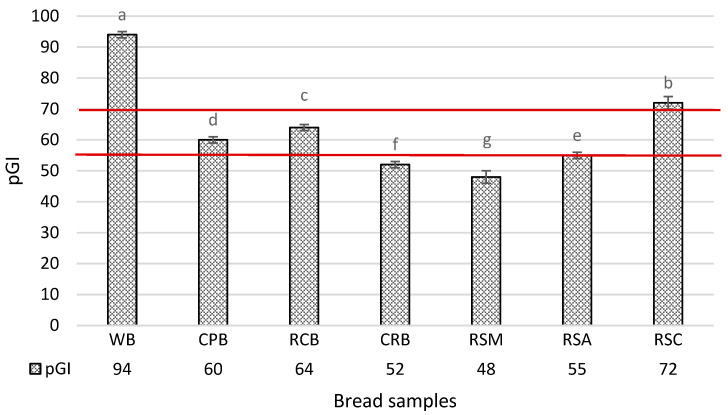
Predicted glycemic index of bread samples. The values are reported below the sample labels. Values with different superscripts are significantly different at *p* < 0.05. pGI: predicted glycemic index; WB: white bread; CPB: bread with 30% chickpea flour; RCB: bread with 10% red chicory powder; CRB: bread with 30% chickpea flour and 10% red chicory powder; RSM: bread with 30% chickpea flour, 10% red chicory powder, and 15% chemically modified tapioca resistant starch; RSA: bread with 30% chickpea flour, 10% red chicory powder, and 15% retrograded tapioca resistant starch; RSC: bread with 30% chickpea flour, 10% red chicory powder, and 15% pregelatinized corn resistant starch. The horizontal lines indicate the values of low GI (up to 55), medium GI (ranging from 56 to 69), and high GI (above 70).

Foods are categorized based on their GI as low (<55), medium (56–69), or high (>70) [18]. White bread is commonly used as a reference food, having a GI value of 100. To develop low GI bread, it is crucial to understand to what extent the inclusion of different ingredients affects the bread’s pGI. The sole incorporation of chickpea flour leads to a pGI reduction of 35.7% compared to the control (Figure 2) and, according to the proposed classification, allows for obtaining medium pGI loaves. The failure to achieve a low pGI bread using chickpea flour alone can be attributed to the interplay of several factors. The low incorporation level of chickpea flour and the small particle size of the flour (leading to an increased starch digestion rate) compared to those of whole chickpeas may have resulted in the lack of a significant difference in the glycemic response, as previously reported by Johnson et al. [19]. However, it is generally recognized that the chickpea flour’s ability to decrease the pGI is because its protein content limits the availability of starch for α-amylase by “encapsulating” it in its structures [20,21]. In addition, legume flours seem to slow carbohydrate absorption due to their content of resistant starch and amylose [22,23]. Previous studies reported that legumes like beans, chickpeas, and lentils moderate the postprandial glucose response, lowering the diet’s GI. Similar results were obtained by Goñi and Valentín-Gamazo and Zafar et al. [14,15] in pasta fortified with chickpea flour. The use of red chicory powder alone reduced the pGI by 31.6%. Polyphenols from red chicory powder could reduce starch digestibility in vitro by inhibiting starch hydrolyzing enzymes. Polyphenols can also interact with starch through non-covalent interactions during cooking, forming starch complexes with partial enzymatic accessibility [11]. Accordingly, Hanhineva et al. [24] demonstrated that different classes of polyphenols can inhibit the in vitro activities of α-amylase and α-glucosidase. Furthermore, polyphenols can limit the rate and degree of starch gelatinization due to hydrogen bonds between phenol and amylose molecules [25]. The same observation was reported by Rocchetti et al. [26] in their studies on fortified bread with grape pomace. 

Nevertheless, the inclusion of red chicory powder allows for obtaining a medium pGI bread. The combination of chickpea flour and red chicory powder further reduces the pGI by 44.1% compared to the control, proving the possibility of exploiting the combined effect of two ingredients to reduce the pGI of bread.

It is widely acknowledged that adding resistant starch can lower a bread’s glycemic index. However, it is crucial to consider that different types of resistant starch and their various origins can affect pGI differently. The main distinctions arise from the resistant starch origins and production methods, as reviewed by Arp et al. [27]. Raigond et al. [9] point out that RS III could be partially and slowly digested in the small intestine. In contrast, RS IV resists enzymatic hydrolysis both in vitro and in the small intestine, as the typical arrangement of starch chains is altered by several substituents (cross-linking) that bind to amylose and amylopectin. Their presence and the resulting spatial changes in the chain can hinder the action of amylolytic enzymes, preventing their regular activity [28]. Highly cross-linked starches lose their swelling abilities during cooking, making their granules less sensitive to enzymes and thus resistant to amylase hydrolysis [29]. Adding RS III from corn in our experimental bread produced a high pGI sample. Instead, RS III from tapioca performed better than the one from corn, and the obtained RSA samples reached a pGI of 55, the upper limit for considering a food with a low glycemic index. The differences observed between the samples made with retrograded resistant starch from corn and tapioca could be attributed to the higher initial content of resistant starch in tapioca flour compared to corn flour, as reported by Martín Bernabé et al. [30]. This observation could also indicate partial starch gelatinization during baking [9].

The best results regarding glycemic index lowering were achieved with chemically modified resistant starch (RS IV), obtained through cross-linking with chemical reagents. Bread prepared with RS IV had a significantly decreased pGI, and it helped classify the obtained bread as a low predicted glycemic index food. The rate and extent of digestion are influenced by a large number of factors, all interconnected, which complicates the understanding of the resistant nature of starch granules [9]. 

The lowering of pGI in the RSM sample with added RS IV is likely due to the chemical modifications and cross-linkages that alter the structure and composition of the starch granules, increasing their resistance to amylolytic enzymes. These modifications, occurring during the industrial process to produce RS IV, create spatial changes in the starch chain that interfere with enzyme access, impeding their regular activity [28].

### 3.2. Bread Technological Properties

The technological properties of control and experimental bread in terms of the moisture content, volume, specific volume, and baking loss (BL) are reported in Table 2. As expected, using different ingredients significantly impacts the technological characteristics of bread.

Overall, experimental bread showed a significant increase in moisture content compared to the control sample due to the fiber addition from chickpea flour, red chicory powder, and RSs that retain water molecules [31].

Additionally, as expected, the substitution of wheat flour with a high dietary fiber ingredient induces a significant decrease in volume and specific volume compared to the control sample. The loss in volume can be attributed to a reduction in the gluten concentration, causing lower gas retention and a concurrent rise in fiber content. Fiber particles can potentially hinder optimal gluten development by intersecting and disrupting gluten strands, thereby inhibiting the formation of a cohesive viscoelastic network and weakening the dough [32,33]. The interaction between dietary fibers and gluten proteins involves complex mechanisms, including changes in disulfide bonds and the secondary structures of the proteins. Indeed, dietary fibers can impact gluten by affecting the α-helix and β-sheet and influencing high-order intermolecular structures through steric hindrance, hydrogen bonding, hydrophobic interactions, and occasionally electrostatic interactions [34]. Moreover, polyphenols from red chicory powder can weaken the gluten strands through their redox reaction on protein disulfide cross-links, thereby reducing the size of the network. As a further effect, polyphenols might reduce the elasticity and impact the final volume of the dough and bread [35].

Furthermore, Saaed et al. [36] reported that roasted chickpea flour lowered the gluten development of biscuits due to water absorption competition between the chickpea components (namely, starches, proteins, and fibers) and wheat protein, thus causing insufficient hydration of the gluten matrix. 

Moreover, the decrease in volume in the new bread formulation could also be ascribed to yeast’s inability to utilize RS during fermentation, which slows down the process [37]. The BL values were higher in the newly formulated bread with RSs than in the control and the other samples without resistant starch. The decrease observed in BL may stem from the abundance of RSs that tend to bind water molecules via hydrogen bonding mechanisms [38].

### 3.3. Texture Profile Analysis

Texture attributes regarding the hardness, cohesiveness, and chewiness are reported in Table 3.

The hardness of the fortified bread increased significantly compared to the control, likely due to reduced gas retention by the gluten network, resulting in a densely packed bread structure [39]. On the contrary, the cohesiveness, which measures the crumb’s internal resistance and its ability to deform before fracturing, decreased in the experimental bread, with the CPB sample exhibiting the highest cohesiveness among the experimental types. A lower cohesiveness indicates a higher susceptibility to rupture, suggesting that fortified bread disintegrates more during chewing and has reduced elasticity and a denser microstructure [40]. A high cohesiveness indicates a better ability to form a bolus rather than crumbling during mastication [34]. The chewiness, reflecting the energy required to masticate food to a ready-to-swallow state, significantly increased with flour substitution. Other studies have also reported that adding dietary fiber significantly increases chewiness in fortified bread [41,42].

### 3.4. Color Analysis

Table 4 reports the results obtained from color analysis using the CIE-LAB colorimetric system for various samples, with parameters of lightness (L*), red/green hue (a*), and yellow/blue hue (b*), as well as the crust and crumb sides of the bread.

The lightness parameter of the crust and crumb significantly decreases in the experimental samples, especially with the substitution with red chicory powder, which changes the color of the samples; the decrease is also visible in the CPB sample, albeit less markedly.

Instead, the a* red index was higher in fortified samples with chicory (RCB, CRB, RSM, RSA, RSC) than in the control due to the high pigmentation of red chicory powder. This redder color is evident at the level of the crumb. On the other hand, the coloration due to the Maillard reaction at the crust level mitigates this effect.

Consequently, for the same reason, the b* yellow index decreases in the crumb and crust in all fortified samples with chicory powder. In the CPB sample, the b* of the crumb increased compared to the control sample due to the typical yellow pigmentation of chickpea flour. 

## 4. Conclusions

The results of this study demonstrate the importance of ingredient selection in producing low-glycemic index bread. Specifically, chickpea flour, red chicory by-product powder, and resistant starch contribute to reducing the glycemic index in bread. The sole inclusion of chickpea flour or red chicory powder reduces the pGI by 35.7% and 31.6%, respectively, while their combination results in a 44.1% reduction. Despite these reductions, the resulting breads still have a medium GI. The use of resistant starch is known to reduce the pGI of foods when incorporated as an ingredient. However, the results highlight how the starch’s type and origin significantly affect this reduction. Bread obtained by adding retrograded RS III from corn produced a high pGI sample. In contrast, the incorporation of retrograded RS III from tapioca had a better performance, giving the bread a pGI of 55, the upper limit value for considering a food with a low glycemic index. Chemically modified RS IV from tapioca significantly decreased the bread’s pGI, classifying the product as a low glycemic index. The lowering of the pGI using RS IV addition was probably due to chemical modifications and cross-linkages that change the structure and composition of the starch granules, increasing their resistance to amylolytic enzymes. Although all the experimental breads are different regarding their technological and texture properties, the one obtained using the retrograded corn resistant starch has a greater impact on both the technological and texture parameters. In conclusion, chickpea flour, red chicory by-product powder, and resistant starch are intriguing ingredients for producing functional bread fortified with dietary fiber. These ingredient selections could contribute to positive effects on human health for the lower predicted glycemic index and, in the end, enhance the economic value of the red chicory food chain with the reuse of a by-product.

## Figures and Tables

**Table 1:** Control and experimental bread formulated with chickpea flour, red chicory powder, and resistant starch.

Sample	Wheat Flour (%)	Chickpea Flour (%)	Red Chicory Powder (%)	Dried Yeast (%)	Sugar (%)	Salt (%)	Guar Gum (%)	Native Gluten (%)	Resistant Starch (%)
WB	86	-	-	2	1	1	5	5	-
CPB	56	30	-	2	1	1	5	5	-
RCB	76	-	10	2	1	1	5	5	-
CRB	46	30	10	2	1	1	5	5	-
RSM	31	30	10	2	1	1	5	5	15
RSA	31	30	10	2	1	1	5	5	15
RSC	31	30	10	2	1	1	5	5	15

WB: white bread; CPB: bread with 30% chickpea flour; RCB: bread with 10% red chicory powder; CRB: bread with 30% chickpea flour and 10% red chicory powder; RSM: bread with 30% chickpea flour, 10% red chicory powder, and 15% chemically modified tapioca resistant starch; RSA: bread with 30% chickpea flour, 10% red chicory powder, and 15% retrograded tapioca resistant starch; RSC: bread with 30% chickpea flour, 10% red chicory powder, and 15% pregelatinized corn resistant starch.

**Table 2:** Technological properties of control and experimental bread with chickpea flour, red chicory powder, and resistant starch.

Sample	Moisture Content (%)	Volume (cm^3^)	Specific Volume (cm^3^/g)	Baking Loss (%)
WB	29.25 ± 1.43 ^d^	1977 ± 8 ^a^	3.53 ± 0.03 ^a^	13.74 ± 0.00 ^b^
CPB	55.70 ± 1.41 ^a^	1863 ± 2 ^c^	3.10 ± 0.09 ^c^	12.24 ± 0.14 ^c^
RCB	47.27 ± 1.16 ^c^	1945 ± 2 ^b^	3.15 ± 0.03 ^b^	11.89 ± 0.10 ^c^
CRB	54.79 ± 0.27 ^a^	1635 ± 2 ^d^	2.58 ± 0.02 ^d^	10.63 ± 0.07 ^d^
RSM	47.30 ± 1.02 ^c^	1626 ± 4 ^e^	2.58 ± 0.08 ^d^	14.58 ± 0.38 ^a^
RSA	51.15 ± 0.11 ^b^	973 ± 2 ^g^	1.55 ±0.05 ^f^	14.89 ± 0.11 ^a^
RSC	47.78 ± 0.25 ^c^	1053 ± 1 ^f^	1.66 ± 0.04 ^e^	14.47 ± 0.36 ^a^

Values with different superscripts within the same column significantly differ at *p* < 0.05. WB: white bread; CPB: bread with 30% chickpea flour; RCB: bread with 10% red chicory powder; CRB: bread with 30% chickpea flour and 10% of red chicory powder; RSM: bread with 30% chickpea flour, 10% red chicory powder, and 15% chemically modified tapioca resistant starch; RSA: bread with 30% chickpea flour, 10% red chicory powder, and 15% retrograded tapioca resistant starch; RSC: bread with 30% chickpea flour, 10% red chicory powder, and 15% pregelatinized corn resistant starch.

**Table 3:** Texture analysis of control and experimental bread with chickpea flour, red chicory powder, and resistant starch.

Sample	Hardness (N)	Cohesiveness (N)	Chewiness (N)
WB	5.70 ± 0.97 ^d^	0.85 ± 0.03 ^a^	4.56 ± 0.92 ^c^
CPB	16.17 ± 3.79 ^c^	0.70 ± 0.04 ^b^	11.20 ± 2.71 ^b^
RCB	20.29 ± 3.32 ^bc^	0.63 ± 0.04 ^b^	12.63 ± 2.64 ^b^
CRB	22.05 ± 2.12 ^b^	0.50 ± 0.08 ^c^	10.90 ± 1.9 ^b^
RSM	23.81 ± 1.92 ^b^	0.46 ± 0.06 ^c^	10.90 ± 0.89 ^b^
RSA	21.40 ± 2.23 ^b^	0.46 ± 0.3 ^c^	9.96 ± 1.24 ^b^
RSC	49.62 ± 1.72 ^a^	0.43 ± 0.06 ^c^	19.65 ± 2.42 ^a^

Values with different superscripts within the same column significantly differ at *p* < 0.05. WB: white bread; CPB: bread with 30% chickpea flour; RCB: bread with 10% red chicory powder; CRB: bread with 30% chickpea flour and 10% red chicory powder; RSM: bread with 30% chickpea flour, 10% red chicory powder, and 15% chemically modified tapioca resistant starch; RSA: bread with 30% chickpea flour, 10% red chicory powder, and 15% retrograded tapioca resistant starch; RSC: bread with 30% chickpea flour, 10% red chicory powder, and 15% pregelatinized corn resistant starch.

**Table 4:** Color analysis of control bread and experimental bread with chickpea flour, red chicory powder, and resistant starch and crust side and crumb side of bread samples.

Sample	Crust				Crumb			
	L*	a*	b*		L*	a*	b*	
WB	51.92 ± 2.99 ^a^	11.94 ± 0.99 ^a^	29.45 ± 2.80 ^a^	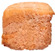	70.85 ± 2.79 ^a^	0.81 ± 0.23 ^e^	17.13 ± 1.12 ^b^	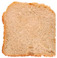
CPB	39.22 ± 2.30 ^b^	13.67 ± 2.17 ^a^	27.61 ± 5.45 ^a^	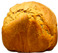	62.00 ± 1.52 ^b^	0.84 ± 0.69 ^d^	26.13 ± 1.70 ^a^	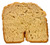
RCB	31.97 ± 5.10 ^c^	12.08 ± 2.58 ^a^	15.66 ± 4.47 ^bc^	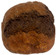	28.63 ± 0.48 ^d^	8.52 ± 0.30 ^b^	10.58 ± 0.69 ^c^	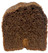
CRB	29.89 ± 2.90 ^cd^	12.34 ± 2.23 ^a^	16.76 ± 3.03 ^b^	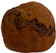	35.88 ± 1.19 ^c^	6.23 ± 0.17 ^c^	10.4 ± 0.59 ^c^	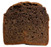
RSM	26.97 ± 2.28 ^d^	12.67 ± 1.75 ^a^	11.76 ± 1.87 ^c^	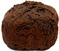	26.29 ± 2.00 ^d^	8.37 ± 0.20 ^b^	9.25 ± 1.60 ^cd^	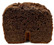
RSA	32.227 ± 2.93 ^c^	12.52 ± 2.00 ^a^	15.65 ± 2.56 ^bc^	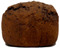	22.72 ± 1.25 ^e^	8.47 ± 0.52 ^b^	8.49 ± 1.04 ^de^	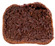
RSC	34.16 ± 4.55 ^c^	12.74 ± 1.67 ^a^	15.76 ± 2.61 ^bc^	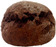	26.30 ± 1.47 ^d^	9.56 ± 0.33 ^a^	7.45 ± 0.92 ^e^	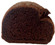

Values with different superscripts within the same column significantly differ at *p* < 0.05. L*: lightness; a*: redness; b*: yellowness; WB: white bread; CPB: bread with 30% chickpea flour; RCB: bread with 10% red chicory powder; CRB: bread with 30% chickpea flour and 10% red chicory powder; RSM: bread with 30% chickpea flour, 10% red chicory powder, and 15% chemically modified tapioca resistant starch; RSA: bread with 30% chickpea flour, 10% red chicory powder, and 15% retrograded tapioca resistant starch; RSC: bread with 30% chickpea flour, 10% red chicory powder, and 15% pregelatinized corn resistant starch.

## Data Availability

The original contributions presented in the study are included in the article, further inquiries can be directed to the corresponding author.
